# Quality Assessment of Solar EUV Remote Sensing Images Using Multi-Feature Fusion

**DOI:** 10.3390/s25206329

**Published:** 2025-10-14

**Authors:** Shuang Dai, Linping He, Shuyan Xu, Liang Sun, He Chen, Sibo Yu, Kun Wu, Yanlong Wang, Yubo Xuan

**Affiliations:** 1 Changchun Institute of Optics, Fine Mechanics and Physics, Chinese Academy of Sciences, Changchun 130033, China; daishuang@ciomp.ac.cn (S.D.);; 2 University of Chinese Academy of Sciences, Beijing 100049, China; 3 College of Communication Engineering, Jilin University, Nanhu Campus, Changchun 130033, China

**Keywords:** image quality assessment, solar physics, deep learning, feature fusion, space weather, FY-3E

## Abstract

**Highlights:**

**What are the main findings?**

- A novel hybrid framework for assessing solar EUV image quality was developed, combining deep learning features from a HyperNet-based model with 22 handcrafted physical and statistical indicators.
- The fusion of these feature types significantly improved the performance of image quality classification, achieving a high accuracy of 97.91% and an AUC of 0.9992.

**What are the implications of the main findings?**

- This method provides a robust and scalable solution for the automated quality con-trol of large-scale solar EUV observation data streams, which is crucial for space weather forecasting.
- The research demonstrates the effectiveness of a multi-feature fusion approach for complex image quality assessment tasks, offering a new direction for similar applica-tions in remote sensing.

**Abstract:**

Accurate quality assessment of solar Extreme Ultraviolet (EUV) remote sensing imagery is critical for data reliability in space science and weather forecasting. This study introduces a hybrid framework that fuses deep semantic features from a HyperNet-based model with 22 handcrafted physical and statistical quality indicators to create a robust 24-dimensional feature vector. We used a dataset of top-quality images, i.e., quality class “Excellent”, and generated a dataset of 47,950 degraded, lower-quality images by simulating seven types of degradation including defocus, blur and noise. Experimental results show that an XGBoost classifier, when trained on these fused features, achieved superior performance with 97.91% accuracy and an AUC of 0.9992. This approach demonstrates that combining deep and handcrafted features significantly enhances the classification’s robustness and offers a scalable solution for automated quality control in solar EUV observation pipelines.

## 1. Introduction

Imaging in the solar Extreme Ultraviolet (EUV) band provides an indispensable observational window into the dynamic processes of the solar atmosphere, enabling critical investigations of space weather drivers such as coronal mass ejections (CMEs) and solar flares [1,2,3]. The Extreme Ultraviolet (EUV) solar telescope, operating in the high-photon-energy EUV band and often using relatively long exposure times, is particularly susceptible to image degradations caused by factors such as scattered contamination particles, mirror surface roughness, platform instability, and increased detector noise [4]. In high-resolution quantitative analyses, such as measurements of coronal loop structures [2], these degradations constitute a critical bottleneck limiting scientific research. Next-generation solar missions, such as Solar Orbiter [5] and Parker Solar Probe [6], and spacecraft on deep-space missions (e.g., spacecraft operating near the Sun-Earth L5 point or on solar orbital trajectories) [7,8] produce a huge amount of data. Their high-resolution imagers take pictures of the Sun very frequently. Over months or years, the total data easily reaches terabytes to petabytes. This creates big challenges for analysis. Automated quality checks and efficient processing are essential to keep the data reliable and useful for science. No-reference image quality assessment (NR-IQA) is an essential technology for prioritizing scientifically viable observations without ground-truth references.

However, the deployment of existing NR-IQA techniques in solar contexts reveals fundamental domain adaptation challenges. Statistical approaches exemplified by **BRISQUE** (Blind/Referenceless Image Spatial Quality Evaluator) [9]—though effective for terrestrial imagery—rely on Generalized Gaussian Distribution (GGD) modeling of locally normalized luminance coefficients. Such priors critically mismatch the radiation physics governing solar EUV imaging, where photon shot noise dominates in low-flux coronal regions and detector point spread functions introduce wavelength-specific blurring artifacts distinct from natural image degradations. Similarly, perceptually motivated methods like VSI [10] fail to capture the scientific saliency of solar features; for instance, their saliency maps often overlook off-limb coronal structures while overemphasizing photospheric granulation patterns irrelevant to coronal physics.

Meanwhile, deep learning-based alternatives [11,12,13,14,15,16], such as HyperIQA [17] and attention-based architectures [18,19], face complementary limitations. Although their content-adaptive networks provide strong nonlinear representation capabilities, GAN-based methods—e.g., the approach proposed by Jarolim et al. [20], which detects anomalies in solar H-alpha filtergrams via image reconstruction and deviation measurement—still rely heavily on large-scale annotated datasets, which are scarcely available in heliophysics.

In contrast, physics-driven quality metrics [21,22,23,24,25], such as the variance of Laplacian (VL) [25], median filter gradient similarity [21], and perceptual measures [24], are widely used to quantify image sharpness. For example, So et al. employed the VL method to assess the clarity of optical solar images [25]. These approaches offer clear physical interpretability but encounter significant challenges in EUV limb observations, where complex edge structures hinder reliable extraction.

Overall, deep learning methods exhibit superior performance but are constrained by data scarcity and their “black-box” nature, whereas purely physics-based approaches provide interpretability but often fail to capture subtle degradations characteristic of coronal imaging. Moreover, deep learning approaches require large-scale expert-labeled datasets, which are fundamentally at odds with typical solar physics practice. Consensus labeling of EUV image quality remains exceptionally challenging due to the following: (1) instrument-specific artifact profiles across different missions, (2) evolving scientific priorities for various solar features, and (3) the lack of standardized degradation metrics tailored to coronal studies. Consequently, directly transferring natural image quality assessment (QA) models to solar EUV images risks mischaracterizing physically significant distortions as negligible noise.

To address these challenges, this study aims to develop and validate a robust and automated quality assessment framework for solar EUV images. The framework is designed to combine deep semantic features with physics-driven statistical features specifically tailored for solar EUV data. In addition, it utilizes a large-scale, realistic simulation dataset based on real solar observations for model training and evaluation. An efficient machine learning classifier is identified to enable potential on-orbit deployment, and the model’s effectiveness is further verified using real on-orbit data, with results interpreted from a solar physics perspective.

To overcome these dual challenges of physical incompatibility and the scarcity of large, expertly annotated solar training datasets, we introduce a hybrid assessment framework that effectively combines deep semantic representations with solar-specific physical diagnostics. Our framework combines deep features with physics-based solar image features. Deep features are patterns that a neural network learns automatically from raw pixel data. They capture complex shapes, structures, and textures in solar images. Handcrafted features, on the other hand, are designed manually using domain knowledge and image processing methods. They include simple measures of texture, sharpness, and noise and directly describe physical properties of solar EUV images. Our method uses the 19.5 nm channel of the Fengyun-3E Solar X-ray and Extreme Ultraviolet Imager (X-EUVI) [26] and introduces two key innovations. First, we use a KonIQ-10k [27] pretrained network as a feature extractor, capturing high-level structural patterns without retraining the entire network. Second, we add physics-based quality metrics that focus on active regions, measuring magnetic feature preservation and thermal signature integrity. By combining these two types of features in a single feature space, our model can perform domain-adaptive quality assessment on labeled solar images.

The objective of this study was to describe and validate a novel method for automatic classification of the quality of solar EUV images.

## 2. Materials and Methods

### 2.1. Data Acquisition from the Detector

The data used in this paper is primarily from the Solar X-ray and Extreme Ultraviolet Imager (X-EUVI) on board the Fengyun-3E (FY-3E) satellite. Since the X-EUVI began its on-orbit operation on 11 July 2021, it has successfully acquired a large volume of solar images and some solar irradiance data. The instrument observes the Sun across multiple channels, including the 19.5 nm extreme ultraviolet (EUV) band and six X-ray channels with wavelength ranges of 0.6–8.0 nm (X1), 0.6–6.0 nm (X2), 0.6–5.0 nm (X3), 0.6–2.0 nm (X4), 0.6–1.6 nm (X5), and 0.6–1.2 nm (X6). These channels probe different temperature regimes of the solar corona, providing a comprehensive view of solar activity and enabling detailed diagnostics of coronal structures and events. The key technical specifications of the back-illuminated CCD detector used by the X-EUVI instrument are summarized in Table 1. It is important to note that these specifications describe the detector itself, which is sensitive to a range of EUV wavelengths. This particular study, however, focuses exclusively on data from the 19.5 nm observation channel.

To ensure the scientific integrity and accuracy of the data, the raw detector data undergoes strict on-orbit processing before release, including geometric correction (to compensate for image deviations caused by attitude changes), noise and dark current removal (subtracting dark frames and suppressing noise), and flat-field calibration (correcting position-dependent detector response to ensure uniform image brightness). After this processing, the data can be used to generate a continuous series of full-disk solar images.

### 2.2. Dataset and ROI Selection Strategy

#### 2.2.1. Image Degradation Simulation and Labeling

The original high-quality FY-3E images are considered “Excellent”. The degradation parameters were designed based on typical quality issues observed in FY-3E solar EUV images, including optical defocus, telescope jitter, detector noise, and local overexposure caused by solar activity. The kernel sizes, noise levels, and overexposure intensities were chosen to realistically simulate the range of conditions encountered during on-orbit observations.

To construct a training and evaluation dataset, we applied seven types of typical degradations, each with five levels (L1–L5), ranging from mild to severe. All kernel sizes and standard deviations are expressed in pixels (px). The seven degradation types are summarized in Table 2.

Through this approach, degraded images corresponding to four quality levels (“Good”, “Moderate”, “Poor”, and “Very Poor”) were generated from the original “Excellent” images, providing a rich and controllable dataset for model training and evaluation. In total, a large-scale dataset of 47,950 images was constructed. Each image was labeled according to a five-level quality standard (Excellent, Good, Moderate, Poor, Very Poor), which served as the target classes for our classification task. The dataset was then split into training and testing sets with an 80%/20% ratio. All images were collected from the 19.5 nm band of the X-EUV imager aboard the FY-3(05) satellite.

#### 2.2.2. Implementation Details

The experiments were conducted on a workstation equipped with an NVIDIA RTX 4060 GPU, a CPU with sufficient computational capacity, and 8 GB of RAM. The software environment includes Python 3.9 and PyTorch version 2023.3.4.

In this experiment, we employed three classical machine learning models for a multi-class classification task: SVM, XGBoost, and Random Forest. For SVM (SVC), probability = True was set to enable probability predictions, and random_state = 42 was fixed, while all other parameters were left at their default values, including the radial basis function (RBF) kernel and unlimited iterations. For XGBoost (XGBClassifier), use_label_encoder = False, eval_metric = ‘logloss’, and random_state = 42 were specified; the learning rate was set to 0.3, the number of trees to 100, and the maximum depth to 6, with all other parameters kept at their defaults. For Random Forest (RandomForestClassifier), only random_state = 42 was set, using the number of trees (100) and unlimited depth. The fixed random seed ensured the reproducibility of the experiments.

In this experiment, we employed three classical machine learning models for a multi-class classification task: SVM, XGBoost, and Random Forest.

Model performance was evaluated using standard metrics including Accuracy, Precision, Recall, F1-score, and AUC (Area Under the Receiver Operating Characteristic Curve). For multi-class classification, Precision, Recall, and F1-score are calculated for each class and then averaged to provide an overall performance measure.

#### 2.2.3. ROI Selection Strategy

**Figure 1:**
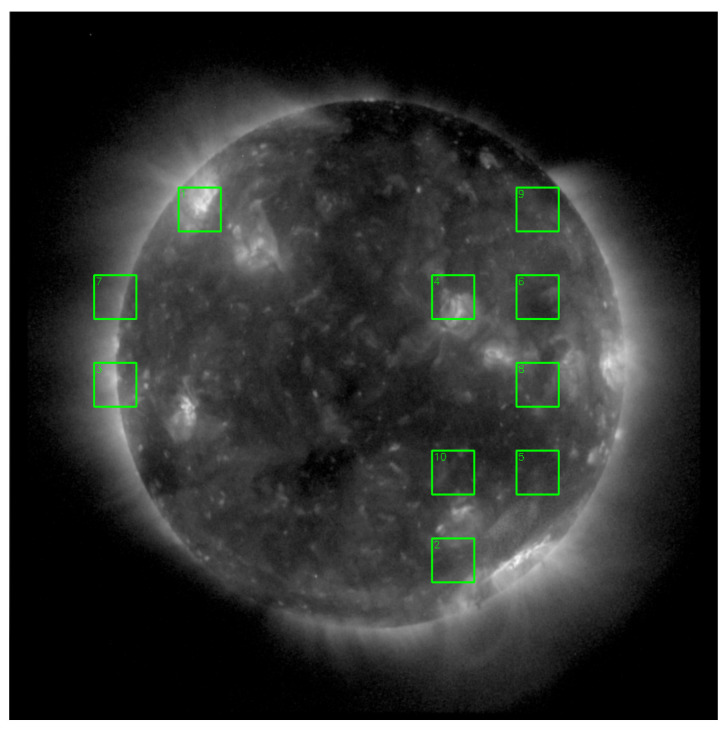
Illustration of the ROI selection, targeting active regions (ARs), limb areas, and other texture-rich zones in a 1024 × 1024 solar EUV image. The green-bordered rectangles indicate the selected ROI (Region of Interest) areas used for feature extraction.

To focus on the most informative parts of the image, we designed an adaptive Region of Interest (ROI) selection strategy. The 1024 × 1024 solar image is divided into 64 × 64 patches. Only patches within the central solar disk are considered. A composite score balancing brightness and gradient is calculated for each patch, and the top 10 patches are selected as ROIs. The process is illustrated in Figure 1.

Brightness score. For patch *i*, the brightness score is defined as

(1) $$B_{k}=\frac{1}{\left|P_{k}\right|}\sum_{(x,y)\in P_{K}}I(x,y)$$

where |*P_k_*| = 64 × 64 is the number of pixels in the patch.

Sharpness score. Sharpness is evaluated via the mean gradient magnitude:

(2) $$S_{k}=\frac{1}{\left|P_{k}\right|}\sum_{\left(x,y\in P_{k}\right)}\sqrt{I_{x}^{2}\left(x,y\right)+I_{y}^{2}\left(x,y\right)}$$

where *I_x_*, *I_y_* are Sobel operators.

Composite ROI score. To balance brightness and sharpness, a weighted composite score is defined:

(3) $$R_{k}=\alpha \frac{B_{k}\;-\;\min(B)}{\max(B)\;-\;\min(B)}\;+\;(1\;-\;\alpha )\frac{S_{k}\;-\;\min(S)}{\max(S)\;-\;\min(S)}$$

where *α* = 0.3 controls the balance between brightness and sharpness, and *B_k_*, *S_k_* are normalized values across all patches. Finally, the top *k* = 10 patches with the highest *R_k_* are selected as ROIs.

### 2.3. Feature Extraction and Fusion

#### 2.3.1. Deep Learning Feature Extraction

To capture high-level semantic information, we employed the backbone network of HyperIQA [17] for deep feature extraction. Specifically, a ResNet-50 architecture [28] pretrained on the KonIQ-10k dataset [27] was used to produce a 112-dimensional global content feature vector, denoted as *f_c_*, for each EUV image. This feature vector represents structural and perceptual quality characteristics.

**Figure 2:**
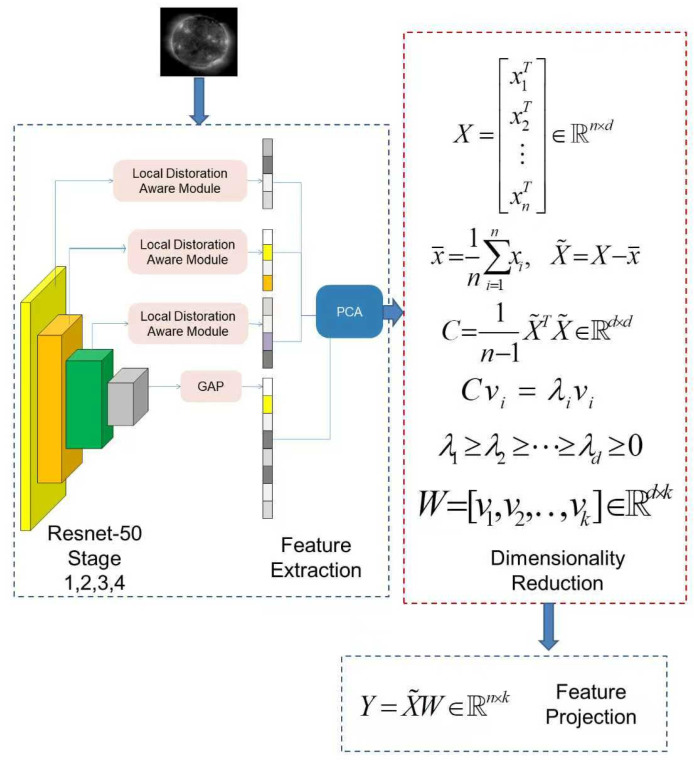
Deep learning feature extraction using HyperIQA. ResNet-50 produces a global content feature vector, which is subsequently reduced by PCA.

To reduce dimensional redundancy, Principal Component Analysis (PCA) [29] was applied to *f_c_*. The top two principal components, explaining more than 95% of the cumulative variance, were retained as the final deep features. This process is illustrated schematically in Figure 2.

#### 2.3.2. Handcrafted Physical–Statistical Features

Complementary to deep features, we designed a set of 22 handcrafted features to quantify the physical and statistical quality attributes of solar EUV images. These features were derived from multiple perspectives, including brightness statistics, sharpness measures, textural properties, noise/fidelity indicators, frequency responses, and spatial descriptors. The categories and representative features are summarized in Table 3. For a complete description of the 22 handcrafted features, including formulas, physical interpretations, and references, see Appendix A.

#### 2.3.3. Feature Fusion and Classification

To validate the complementarity of the extracted features, three comparative experimental settings were designed:

Deep features only (2-dimensional, PCA-reduced);

Handcrafted features only (22-dimensional);

Fused features (24-dimensional), constructed by concatenating the deep and handcrafted feature vectors.

**Figure 3:**
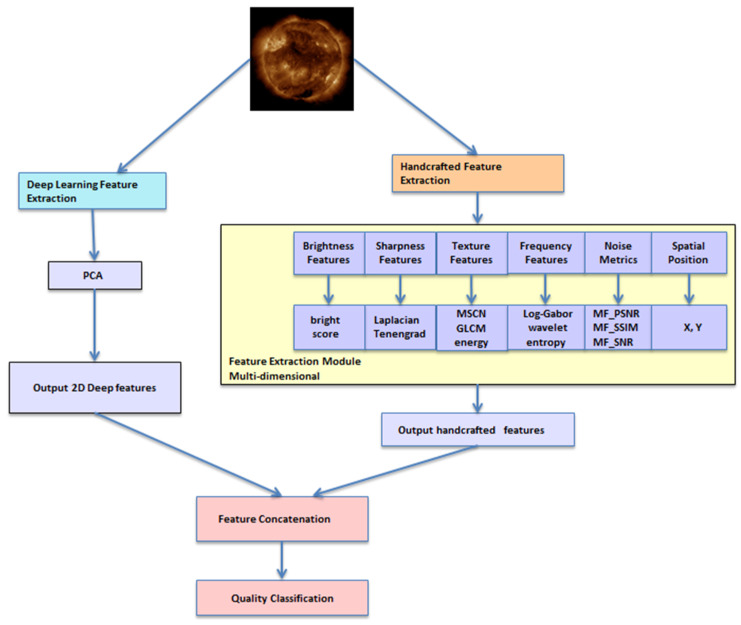
Overview of the feature extraction and fusion framework. Handcrafted physical features and deep semantic features are concatenated into a fused feature vector for classification.

The three feature sets were evaluated using representative machine learning classifiers: Support Vector Machine (SVM) [38], XGBoost [39], and Random Forest [40]. The complete pipeline for feature extraction, fusion, and classification is presented in Figure 3.

## 3. Results

### 3.1. Performance Analysis

The performance of different feature–classifier combinations was systematically evaluated, and the results are summarized in Table 4.

The results indicate that the XGBoost classifier combined with fused features achieved the highest performance, with an accuracy of 97.91% and an AUC of 0.9992. Handcrafted features alone also yielded strong results with tree-based classifiers, achieving 97.50% accuracy using XGBoost. Deep features alone provided moderate performance, with Random Forest achieving 79.51% accuracy. SVM consistently exhibited lower performance across all feature sets.

**Figure 4:**
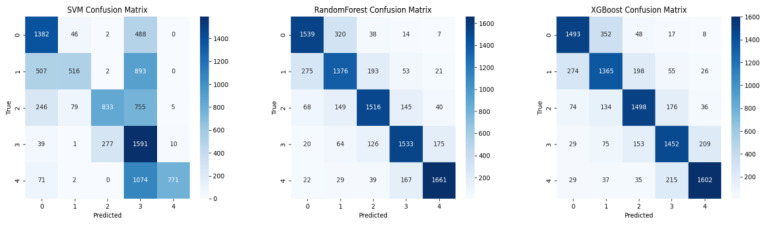
Confusion matrices for deep features only with three classifiers: SVM, XGBoost, and Random Forest (Accuracy: 77.27%).

**Figure 5:**
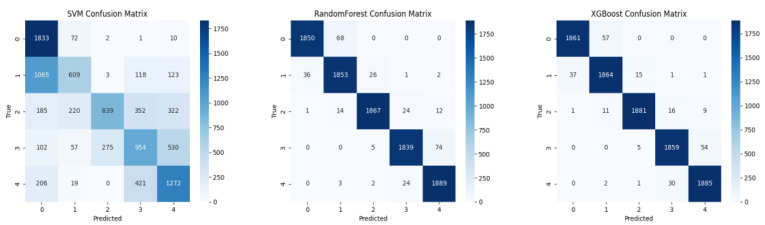
Confusion matrices for handcrafted features only (22D) with three classifiers: SVM, XGBoost, and Random Forest (Accuracy: 97.50%).

**Figure 6:**
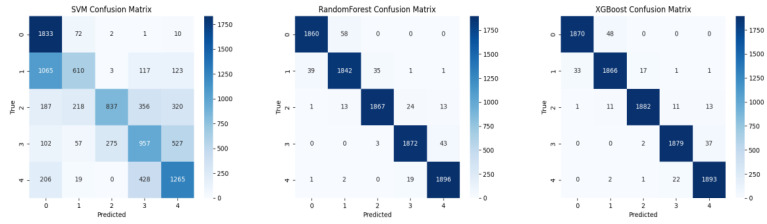
Confusion matrices for fused features (22 handcrafted + 2 deep features) with three classifiers: SVM, XGBoost, and Random Forest (Accuracy: 97.91%).

The confusion matrices using each feature set are presented in Figure 4, Figure 5 and Figure 6.

### 3.2. Feature Analysis

The dominance of handcrafted physical features is unequivocal: with XGBoost, the 22 solar-specific metrics alone achieved 97.50% accuracy, underscoring their capacity to encode fundamental coronal quality determinants—from active region texture integrity to off-limb sharpness degradation. This exceptional performance confirms that solar EUV image quality can be effectively quantified through physics-driven metrics. These metrics are designed to capture critical quality determinants such as the integrity of thermal signatures during flares. They also capture the presence of instrument-specific noise patterns in coronal holes. Although feature fusion yielded a marginal accuracy gain (0.41%), this increment proves operationally critical. As Figure 6 verifies, fused features improved the recall for the ‘Very Poor’ class (Level 5) (these images are typically considered scientifically unusable) recall by 0.42% (98.2% → 98.6%), directly enhancing downstream data utility for flare forecasting pipelines. This precision in identifying irrecoverably degraded images is paramount for downlink optimization, preventing bandwidth waste on unusable data, event detection, ensuring viable CME/flare analysis frames, long-term monitoring, and maintaining calibration consistency.

### 3.3. Ablation Study

To evaluate the contribution of different features in the FY-3E X-EUV 19.5 nm image quality assessment task, we conducted ablation experiments using the XGBoost classifier and reported the classification results for the five quality levels (L1–L5).

Deep Features (HyperIQA PCA 2D)**:** The overall accuracy was 77.3% with an F1 score of 0.773. The per-class accuracy indicates notable confusion in the middle levels (L2–L4). For instance, approximately 14.5% of L2 samples were misclassified as L1, and about 10.8% of L4 samples were misclassified as L5, suggesting that deep features alone are insufficient to effectively distinguish intermediate quality levels.

Handcrafted Features (22D): The overall accuracy increased significantly to 97.5% with an F1 score of 0.975. All classes exhibited stable performance, with almost no confusion among L2–L4. For example, the misclassification rate of L3 was below 1%, while L4 had a small portion misclassified as L5 (~2.8%). These results indicate that the handcrafted physical–statistical features possess strong discriminative power for image quality assessment.

Fused Features (22 + 2D)**:** The overall accuracy further improved to 97.9% with an F1 score of 0.979. The fused features achieved noticeably better classification performance for the intermediate levels. For example, misclassifications in L3–L4 were further reduced, and the misclassification rate of L5 decreased to about 1.1%. This demonstrates that combining deep and handcrafted features can effectively exploit complementary information, thereby enhancing XGBoost’s discriminative ability across all image quality levels.

In summary, the ablation study shows that handcrafted features dominate the FY-3E image quality assessment task, while the addition of deep features further improves the classification of intermediate levels, achieving more precise five-level quality prediction.

### 3.4. On-Orbit Measurement Verification and Solar Physics Interpretation

To validate the applicability and physical relevance of the proposed image quality assessment framework in actual space observation missions, this paper selects and analyzes image data from the 19.5 nm channel of the X-EUVI instrument aboard the Fengyun-3E (FY-3E) satellite. This section not only tests the model’s evaluation capability on real images but also explores the potential impact of image degradation on solar physics observations.

#### 3.4.1. Normal Observation Images and Stable Physical Features

**Figure 7:**
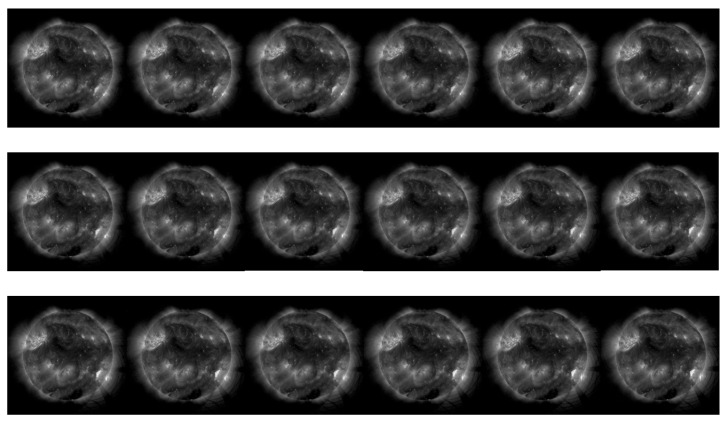
A sequence of normal observation images, with a 7-s interval between each image download and an exposure time of 800 ms (a typical observational sequence).

Figure 7 displays a sequence of routine observation images with no significant distortion, all of which were predicted as Grade 1 quality. Figure 7 shows the physical feature curves extracted from this image sequence, including mean brightness, contrast, Mean Subtracted Contrast Normalized (MSCN) consistency, and log-Gabor response, all of which remain relatively stable. In this type of image, active region structures are clear, and details of coronal loops and bright points are rich, making them suitable for Differential Emission Measure (DEM) temperature inversion and magnetic field evolution analysis. The image quality assessment model successfully identified these high-quality images, demonstrating its capability to preserve scientifically valid data.

**Figure 8:**
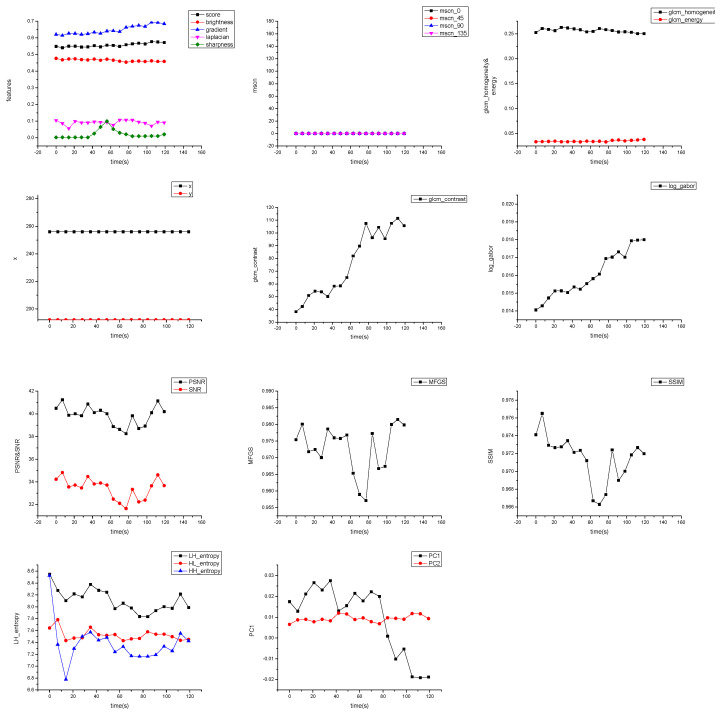
Extracted results of image physical features: brightness, contrast, MSCN, and log-Gabor energy.

From Figure 8, it can be observed that the mean brightness of the image sequence is fundamentally stable throughout the observation period with minimal fluctuation, indicating a good exposure status of the imager and no significant solar irradiation changes. The image contrast also remains at a relatively uniform level, suggesting that the image edges are sharp and there is no obvious degradation in structural acuity.

The experimental results indicate that the image quality metrics exhibit only minor fluctuations, as shown in Table 5.

Further observation reveals that although the MSCN consistency coefficient is generally stable, it exhibits slight fluctuations. This may be related to changes in local image texture density during the observation process, such as dynamic evolution of structures at the edges of active regions causing local texture variations. The log-Gabor energy shows minor fluctuations, which is presumed to be associated with short-term changes in localized bright areas (e.g., coronal bright spots, faint pre-flare structures). However, the overall trend does not show a systematic decline, indicating that the images still retain key mid-to-high frequency texture structures. The observed increasing trends in GLCM-contrast and log-Gabor energy suggest a progressive enhancement in the structural richness and high-frequency content of the image sequence, which may indicate intensified solar activity or improved imaging clarity. However, the potential influence of noise should also be considered.

In summary, all physical features of this image set demonstrate strong consistency and stability, confirming that their image quality is at an optimal level. They possess the capability to fully preserve high-value information such as solar coronal structures and active region contours, supporting their use in scientific-grade data analysis tasks like DEM temperature reconstruction and magnetic flux evolution studies.

#### 3.4.2. Exposure Variation and Image Photometric Attenuation

**Figure 9:**
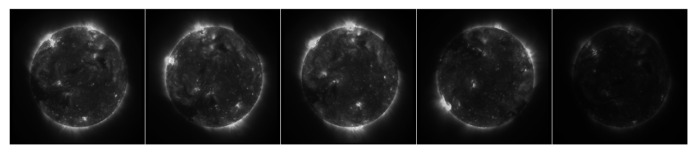
Schematic diagram of the effect of exposure time variation on image quality (the exposure times from left to right are 2400 ms, 3600 ms, 3600 ms, 3600 ms, and 800 ms, respectively; image content displays a sequence of solar images with decreasing brightness).

**Figure 10:**
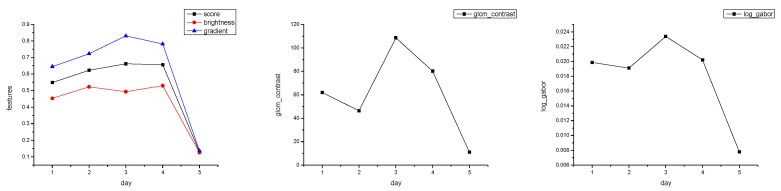
Corresponding changes in physical feature curves: decrease in brightness and contrast. The maximum fluctuations of the score, brightness, and gradient metrics reached 0.7, 0.3, and 0.5, respectively, which are significantly higher than those shown in Figure 8 (where fluctuations did not exceed 0.1). For texture features, the GLCM contrast exhibited a maximum fluctuation exceeding 90, compared to less than 80 in Figure 8. Similarly, the log-Gabor feature fluctuated by 0.016, which is substantially higher than the ≤0.005 variation observed in Figure 8.

Figure 9 presents a sequence of images showing brightness attenuation caused by changes in exposure parameters. The images correspond to exposure times of 2400 ms, 3600 ms (×3), and 800 ms, with predicted quality grades of 2, 2, 2, 2, and 3, respectively. Figure 10 shows the corresponding extracted physical features, where brightness and contrast decrease significantly. By comparison with GOES X-ray flux data and ruling out flare events, it is further inferred that the signal attenuation may be caused by imager aging or filter contamination. This type of degradation could affect the detection and classification of faint active regions, thereby impacting the a priori judgment of flare eruptions.

#### 3.4.3. Field-of-View Deviation

**Figure 11:**
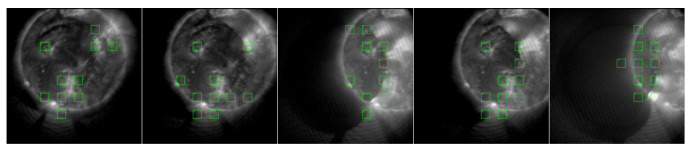
A sequence of solar images under conditions of pointing offset (image content displays a sequence of solar images drifting towards the edge of the frame).

Figure 11 showcases a set of on-orbit images where the sun has deviated from the center of the field of view. The predicted quality grades are 1, 1, 1, 1, and 2. This phenomenon occurs because the satellite’s three-axis attitude cannot maintain a stable alignment with the sun at certain times due to orbital constraints. The model did not significantly lower its quality score, primarily because global offset types of distortion were not introduced into the training set, and the Region of Interest (ROI) selection was focused on local areas.

**Figure 12:**
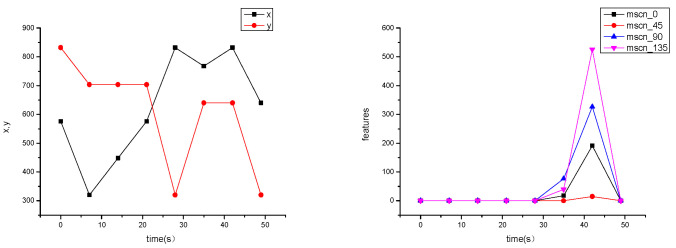
Drastic fluctuations in ROI coordinates and MSCN, indicating non-natural change factors. The spatial coordinates exhibited fluctuations exceeding (500, 500), and the MSCN features along three directions fluctuated by more than 100, far surpassing the values reported in Figure 8.

In Figure 12, supplementary features we extracted—ROI coordinate changes and MSCN fluctuations—exhibit drastic jumps within a very short period (50 s). In comparison with the spatial location of the ROI and MSCN in Figure 8, this is distinct from the change cycle of coronal activity and suggests the possible existence of platform thermal deformation or mechanical pointing anomalies.

### 3.5. Cross-Dataset Validation Considerations

It should be noted that, to date, there is no publicly available EUV image dataset with systematically annotated degradation levels. The solar dynamics observatory atmospheric imaging assembly (SDO/AIA) [41] subsets (e.g., 193 Å) mainly consist of standard-calibrated high-quality observations and lack annotations for degradation levels or quality metrics, making them unsuitable for direct cross-dataset validation. In addition, images from SDO/AIA wavelengths exhibit highly consistent instrument characteristics, spatial resolution, and preprocessing procedures. Therefore, a model trained on one subset is expected to show limited performance variation on other subsets. Based on these considerations, the experiments conducted in this study are sufficient to demonstrate the robustness and generalization capability of the proposed model.

## 4. Discussion

Our analysis demonstrates that the proposed hybrid feature-fusion framework provides a robust and practical approach for assessing solar EUV image quality. Handcrafted, physics-driven features achieved high accuracy, while the integration with deep semantic features offered a modest yet meaningful improvement, capturing structural patterns that may be overlooked by purely physical metrics. The choice of classifier further enhanced performance: XGBoost outperformed SVM in both accuracy and inference speed, making it particularly suitable for near real-time on-orbit deployment. On-orbit validation confirmed that the model can reliably distinguish high-quality observations from degraded images caused by operational factors.

Previous studies have explored deep learning-based methods and physics-driven quality metrics [6,33,34,35,36,37,38,39,40,41], which face limitations in data availability, interpretability, or performance on EUV limb observations. In contrast, our hybrid feature-fusion approach achieves high accuracy while maintaining physical interpretability and computational efficiency.

The proposed method works well for extracting local image features and assessing degradation, but it is not sensitive to global shifts. If an image is systematically translated or rotated, the model may not capture the overall position change, which can affect some quality metrics or physical measurements. The current method also analyzes single frames and does not fully use temporal information from multiple frames, which may limit its performance on fast-changing or transient events. Future work could include adding global alignment or shift compensation, such as image registration or Spatial Transformer Networks (STNs), and using multi-frame temporal modeling with LSTM or Transformer to analyze consecutive frames and improve robustness to global shifts and transient changes.

## 5. Conclusions

This study successfully developed and validated a novel framework for solar EUV image quality assessment by fusing deep semantic features with domain-specific, physical–statistical features. This hybrid approach, particularly when paired with an XGBoost classifier, achieved good performance with 97.91% accuracy. While well-designed handcrafted features are extremely powerful, their fusion with deep features provides a crucial performance boost, especially in identifying the most severely degraded images. This work provides an effective and robust solution for the automated quality classification of massive solar datasets, holding significant promise for operational space weather forecasting and future deep space missions.

## Figures and Tables

**Table 1:** Main technical specifications of CCD on FY-3E.

Parameter	Value
Type	Back-illuminated, frame transfer
Average quantum efficiency	45%
Pixel resolution	1024 × 1024
Pixel size	13 μm × 13 μm
Peak full well capacity	100 ke^−^
Output responsivity	3.5 μV/e^−^
Readout noise	8 e^−^ rms (1.33 MHz)
Output ports	2

**Table 2:** Degradation types and parameter settings for solar EUV images.

Degradation Type	L1	L2	L3	L4	L5
Defocus Blur	Radius 3	Radius 5	Radius 9	Radius 13	Radius 17
Motion Blur	3 @ 0°	5 @ 30°	9 @ 45°	13 @ 60°	17 @ 90°
Gaussian Blur	3, *σ* = 0.5	5, *σ* = 1.0	9, *σ* = 2.0	13, *σ* = 3.0	17, *σ* = 4.0
Gaussian Noise	Var 0.0005	Var 0.001	Var 0.005	Var 0.01	Var 0.02
Salt–Pepper Noise	Prob 0.0005	Prob 0.001	Prob 0.005	Prob 0.01	Prob 0.02
Mixed Blur+Noise*	D3 + M3 @ 0° + G3,σ0.5 + N (G0.0005/S&P0.0005)	D5 + M5 @ 30° + G5,σ1.0 + N (G0.001/S&P0.001)	D9 + M9 @ 45° + G9,σ2.0 + N (G0.005/S&P0.005)	D13 + M13 @ 60° + G13,σ3.0 + N (G0.01/S&P0.01)	D17 + M17 @ 90° + G17,σ4.0 + N (G0.02/S&P0.02)
Overexposure	+10	+15	+20	+30	+40

Notes. Radius denotes the kernel radius (pixels), *σ* is the standard deviation of Gaussian blur, Var represents the variance of Gaussian noise, and Prob indicates the probability of salt-and-pepper noise. Abbreviations used in mixed degradation include D for defocus blur, M for motion blur, G for Gaussian blur, and N for noise (Gaussian/Salt–Pepper).

**Table 3:** Categories of handcrafted features employed in solar EUV image quality assessment.

Category	Features	References
Brightness	Activity score, mean intensity	[30]
Sharpness	Mean gradient, Laplacian, Tenengrad	[31]
Texture	MSCN coefficient consistency; GLCM contrast, homogeneity, energy	[9,32]
Noise/Fidelity	MFGS; PSNR, SSIM, SNR vs. median-filtered version	[33,34,35]
Frequency	Log-Gabor responses, wavelet entropy	[36]
Spatial	Patch coordinates	[37]

Notes: MSCN (Mean Subtracted Contrast Normalization), GLCM (Gray-Level Co-occurrence Matrix), MFGS (Median Filter Gradient Similarity), PSNR (Peak Signal-to-Noise Ratio), SSIM (Structural Similarity Index), and SNR (Signal-to-Noise Ratio) are standard image quality assessment metrics used in this study.These handcrafted features are specifically tailored to solar imaging conditions, capturing degradations induced by photon shot noise, instrument-specific blur, and the preservation of coronal structures.

**Table 4:** Comparative classification performance for FY-3E X-EUV 19.5 nm image quality assessment.

Feature Type	Classifier	Accuracy	Precision	Recall	F1 Score	AUC	Training Time (s)	Prediction Time (s)
Deep Features (HyperIQA PCA 2D)	SVM	0.5311	0.6954	0.5311	0.5320	0.8328	161.11	21.85
	XGBoost	0.7727	0.7734	0.7727	0.7730	0.9641	0.66	0.015
	Random Forest	0.7951	0.7954	0.7951	0.7952	0.9691	6.65	0.208
Handcrafted Features (22D)	SVM	0.5742	0.5988	0.5742	0.5560	0.8887	209.34	29.65
	XGBoost	0.9750	0.9751	0.9750	0.9750	0.9990	1.49	0.024
	Random Forest	0.9696	0.9697	0.9696	0.9696	0.9985	10.68	0.146
Fused Features (22 + 2D)	SVM	0.5737	0.5986	0.5737	0.5556	0.8885	214.38	30.93
	XGBoost *	**0.9791**	**0.9792**	**0.9791**	**0.9792**	**0.9992**	**1.25**	**0.024**
	Random Forest	0.9736	0.9736	0.9736	0.9736	0.9988	10.75	0.157

Notes. SVM(Support Vector Machine); * Among all classifiers, XGBoost combined with fused features achieved the best performance, with an accuracy of 97.91% and an AUC of 0.9992.

**Table 5:** Observed variations in image features in FY-3E solar EUV dataset.

Feature	Observed Variation
Low-level metrics (score, brightness, gradient, Laplacian, sharpness)	≤0.1 (normalized units)
PSNR(Peak Signal-to-Noise Ratio)	±3–4 dB
SNR (Signal-to-Noise Ratio)	±3–4 dB
GLCM-based texture features	< 0.01
Spatial coordinates	Stable (no geometric misregistration)
Overall contrast (Michelson contrast)	80
Log-Gabor energy	0.004
MFGS (median filter–gradient similarity)	≤0.025
SSIM (structural similarity index)	<0.011
HH entropy (high-frequency entropy)	1.8
PC1 (first principal component)	≤0.05

## Data Availability

The code is available at: https://github.com/liliansnail/EUV-Image-Quality (accessed on 20 September 2025).

## Appendix A

**Table A1:** Feature definitions for solar EUV image quality evaluation.

Feature	Definition	Solar EUV Image Meaning
brightness	Mean brightness of the image block	Indicates local radiative intensity; higher brightness corresponds to heated coronal regions.
score	Normalized image quality score	Combines brightness and gradient, reflecting both illumination and structural activity of solar regions.
gradient	Average gradient magnitude of the block	Highlights edges such as coronal loop boundaries, filaments, or CME fronts.
laplacian	Variance of the Laplacian (focus measure)	Sensitive to small-scale variations, revealing fine coronal structures.
sharpness	Sharpness based on edge strength	High sharpness indicates well-resolved loop systems or active region cores.
mscn_0	MSCN coefficient in 0° direction	Captures local structural correlation along horizontal direction.
mscn_45	MSCN coefficient in 45° direction	Captures local structural correlation along diagonal direction (45°).
mscn_90	MSCN coefficient in 90° direction	Captures local structural correlation along vertical direction.
mscn_135	MSCN coefficient in 135° direction	Captures local structural correlation along diagonal direction (135°).
glcm_contrast	GLCM contrast feature	High contrast values reveal strong magnetic neutral lines or flare kernels.
glcm_homogeneity	GLCM homogeneity feature	Quiet Sun regions show high homogeneity; active regions show lower values.
glcm_energy	GLCM energy feature	Indicates textural regularity; high energy corresponds to repetitive loop structures.
log_gabor	Log-Gabor filter response	Captures multi-scale curved structures, useful for identifying CME fronts or loops.
MFGS	Median Filter Gradient Similarity metric	A solar-specific focus measure; evaluates clarity of coronal fine structures.
PSNR	Peak Signal-to-Noise Ratio	Reflects overall fidelity of the image, useful for quantifying degradation effects.
SSIM	Structural Similarity Index	Sensitive to luminance, contrast, and structural changes in solar features.
SNR	Signal-to-Noise Ratio	Indicates detectability of faint eruptions or coronal dimmings against background noise.
LH_entropy	Wavelet entropy of LH sub-band	Reflects complexity of horizontal structures (e.g., plasma flows).
HL_entropy	Wavelet entropy of HL sub-band	Reflects complexity of vertical structures (e.g., coronal loops).
HH_entropy	Wavelet entropy of HH sub-band	Reflects diagonal structural complexity, often linked to turbulence.
x	x-coordinate of the upper-left corner of the block	Preserves spatial context, showing whether the region lies near disk center or limb.
y	y-coordinate of the upper-left corner of the block	Preserves spatial context, showing whether the region lies near disk center or limb.

(A1)
$$\mathrm{brightness}=\frac{1}{N}\sum_{i,j}I(i,j)$$

(A2)
$$\mathrm{gradient}=\frac{1}{N}\sum_{i,j}\sqrt{G_{x}{\left(i,j\right)}^{2}+G_{y}{\left(i,j\right)}^{2}}$$

(A3)
score=α⋅brightness+(1−α)⋅gradient

(A4)
$$L=\nabla^{2}I=\frac{\partial^{2}I}{\partial x^{2}}+\frac{\partial^{2}I}{\partial y^{2}}$$

(A5)
$$\mathrm{laplacian}=\frac{1}{\left|M\right|}\sum_{(i,j)\in M}|L|$$

(A6)
$$T_{k}=G_{x}^{2}+G_{y}^{2},\;\mathrm{sharpness}=\frac{1}{K}\sum_{k=1}^{K}\mathrm{var}(T_{k})$$

(A7)
$$\hat{I}(x,y)=\frac{I(x,y)-\mu (x,y)}{\sigma (x,y)+\gamma }$$

(A8)
$${\mathrm{MSCN}}_{\mathrm{product},\theta }=\hat{I}(x,y)⊗\hat{I}(x^{\prime },y^{\prime })$$

(A9)
$$\mathrm{glcm}\_\mathrm{contrast}=\sum_{i,j}{\left(i-j\right)}^{2}P(i,j)$$

(A10)
$$\mathrm{glcm}\_\mathrm{homogeneity}=\sum_{i,j}\frac{P(i,j)}{1+|i-j|}$$

(A11)
$$\mathrm{glcm}\_\mathrm{energy}=\sum_{i,j}P{\left(i,j\right)}^{2}$$

(A12)
$$\mathrm{MFGS}=1-\frac{\sum {\left(G(I)-G(I_{\mathrm{median}})\right)}^{2}}{\sum G{\left(I\right)}^{2}}$$

(A13)
$$\mathrm{PSNR}=10\log_{10}\frac{MAX_{I}^{2}}{\mathrm{MSE}}$$

(A14)
$$\mathrm{SSIM}=\frac{\left(2\mu_{x}\mu_{y}+C_{1})(2\sigma_{xy}+C_{2}\right)}{\left(\mu_{x}^{2}+\mu_{y}^{2}+C_{1})(\sigma_{x}^{2}+\sigma_{y}^{2}+C_{2}\right)}$$

(A15)
$$\mathrm{SNR}=\frac{\mathrm{mean}(\mathrm{signal})}{\mathrm{std}(\mathrm{noise})}$$

(A16)
$$G(u,v)=\exp\left(-\frac{{\left[\log\left(\frac{\sqrt{u^{2}+v^{2}}}{f_{0}}\right)\right]}^{2}}{2{\left(\log\sigma \right)}^{2}}\right),\;G(0,0)=0$$

(A17)
$$entropy=-\sum_{i}p_{i}\log_{2}(p_{i})$$

Equation (A17) where $p_{i}$ denotes the probability distribution of wavelet coefficients. Although the mathematical expression is identical, the features are computed on different wavelet sub-bands (LH, HL, and HH, respectively), thereby capturing distinct directional and frequency characteristics of the solar EUV images.

*x*-coordinate of the upper-left corner of the block

*y*-coordinate of the upper-left corner of the block

The definitions of the symbols in the above formulas are as follows:

I(x,y): pixel intensity at spatial coordinate (x,y)

*μ*: mean intensity of the block (local mean)

σ: standard deviation of intensity in the block

∇I: image gradient (computed by Sobel operator)

ΔI: Laplacian of the image (second-order derivative)

Var(ΔI): variance of Laplacian, used for sharpness

Gx,Gy: gray-level co-occurrence matrix (GLCM) probability of intensity pai

(i,j): are the horizontal and vertical image gradients

$\hat{I}(i,j)$: normalized MSCN coefficient at pixel (i,j)

γ: stabilizing constant to avoid division by zero in MSCN

⊗: element-wise product between neighboring MSCN coefficients

θ: orientation of MSCN products (0°, 45°, 90°, 135°)

p(i,j): the probability of the gray-level pair (i,j) occurring in the GLCM

G: gradient similarity index (MFGS metric)

MSE: mean squared error between original and degraded block

C1,C2: A small constant to avoid division by zero

G(u,v): log-Gabor filter response, u,v: frequency domain coordinates,

$f_{0}$: center frequency

σ: parameter controlling the bandwidth, G (0,0): used to avoid the DC component

## References

[1] Benz A.O. (2017). Flare observations. Living Rev. Sol. Phys..

[2] Temmer M. (2021). Space weather: The solar perspective. Living Rev. Sol. Phys..

[3] Yashiro S., Gopalswamy N., Michalek G., St. Cyr O.C., Plunkett S.P., Rich N.B., Howard R.A. (2004). A Catalog of White Light Coronal Mass Ejections Observed by the SOHO Spacecraft. J. Geophys. Res. Space Phys..

[4] BenMoussa A., Gissot S., Schühle U., Del Zanna G., Auchere F., Mekaoui S., Jones A.R., Dammasch I.E., Deutsch W., Dinesen H. (2013). On-orbit degradation of solar instruments. Sol. Phys..

[5] Müller D., St. Cyr O.C., Zouganelis I., Gilbert H.R., Marsden R., Nieves-Chinchilla T., Antonucci E., Auchere F., Berghmans D., Horbury T.S. (2020). The Solar Orbiter mission—Science overview. Astron. Astrophys..

[6] Raouafi N.E., Matteini L., Squire J., Badman S.T., Velli M., Klein K.G., Chen C.H.K., Whittlesey P.L., Laker R., Horbury T.S. (2023). Parker Solar Probe: Four years of discoveries at solar cycle minimum. Space Sci. Rev..

[7] Zhu Z.M., Leng X.Y., Guo Y., Li C., Li Z., Lu X., Huang F., You W., Deng Y., Su J. (2025). Research on the principle of multi-perspective solar magnetic field measurement. Res. Astron. Astrophys..

[8] Deng Y., Zhou G., Dai S., Wang Y., Feng X., He J., Jiang J., Tian H., Yang S., Hou J. (2023). Solar Polar Orbit Observatory. Sci. Bull..

[9] Mittal A., Moorthy A.K., Bovik A.C. (2012). No-reference image quality assessment in the spatial domain. IEEE Trans. Image Process..

[10] Zhang L., Shen Y., Li H. (2015). VSI: A visual saliency-induced index for perceptual image quality assessment. IEEE Trans. Image Process..

[11] Yang X., Li F., Liu H. (2019). A survey of DNN methods for blind image quality assessment. IEEE Access.

[12] Agnolucci L., Galteri L., Bertini M., Del Bimbo A. Arniqa: Learning distortion manifold for image quality assessmentt. Proceedings of the 2024 IEEE/CVF Winter Conference on Applications of Computer Vision (WACV).

[13] Chiu T.-Y., Zhao Y., Gurari D. Assessing Image Quality Issues for Real-World Problems. Proceedings of the 2020 IEEE/CVF Conference on Computer Vision and Pattern Recognition (CVPR).

[14] Madhusudana P.C., Birkbeck N., Wang Y., Adsumilli B., Bovik A.C. Image quality assessment using contrastive learning. Proceedings of the 2022 IEEE/CVF Winter Conference on Applications of Computer Vision Workshops (WACVW).

[15] Golestaneh S.A., Dadsetan S., Kitani K.M. No-reference image quality assessment via transformers, relative ranking, and self-consistency. Proceedings of the 2022 IEEE/CVF Winter Conference on Applications of Computer Vision (WACV).

[16] Kang L., Ye P., Li Y., Doermann D. Convolutional neural networks for no-reference image quality assessment. Proceedings of the IEEE Conference on Computer Vision and Pattern Recognition.

[17] Su S., Yan Q., Zhu Y., Zhang C., Ge S., Sun W., Zhang Y. Blindly assess image quality in the wild with a content-aware hypernetwork. Proceedings of the 2020 IEEE/CVF Conference on Computer Vision and Pattern Recognition(CVPR).

[18] Wang C., Lv X., Fan X., Ding W., Jiang X. (2023). Two-channel deep recursive multi-scale network based on multi-attention for no-reference image quality assessment. Int. J. Mach. Learn. Cybern..

[19] Yang S., Wu T., Shi S., Lao S., Gong Y., Cao M., Wang J., Yang Y. Maniqa: Multi-dimension attention network for no-reference image quality assessment. Proceedings of the 2022 IEEE/CVF Conference on Computer Vision and Pattern Recognition Workshops (CVPRW).

[20] Jarolim R., Veronig A.M., Pötzi W., Podladchikova T. (2020). Image-quality assessment for full-disk solar observations with generative adversarial networks. Astron. Astrophys..

[21] Deng H., Zhang D., Wang T., Liu Z., Xiang Y., Jin Z., Cao W. (2015). Objective image-quality assessment for high-resolution photospheric images by median filter-gradient similarity. Sol. Phys..

[22] Popowicz A., Radlak K., Bernacki K., Orlov V. (2017). Review of image quality measures for solar imaging. Sol. Phys..

[23] Denker C., Dineva E., Balthasar H., Verma M., Kuckein C., Diercke A., Manrique S.J.G. (2018). Image quality in high-resolution and high-cadence solar imaging. Sol. Phys..

[24] Huang Y., Jia P., Cai D., Cai B. (2019). Perception evaluation: A new solar image quality metric based on the multi-fractal property of texture features. Sol. Phys..

[25] So C.W., Yuen E.L.H., Leung E.H.F., Pun J.C.S. (2024). Solar image quality assessment: A proof of concept using variance of Laplacian method and its application to optical atmospheric condition monitoring. Publ. Astron. Soc. Pac..

[26] Chen B., Ding G.X., He L.P. (2022). Solar X ray and Extreme Ultraviolet Imager (X EUVI) loaded onto China’s Fengyun 3E satellite. Light Sci. Appl..

[27] Hosu V., Lin H., Sziranyi T., Saupe D. (2020). KonIQ-10k: An ecologically valid database for deep learning of blind image quality assessment. IEEE Trans. Image Process..

[28] He K., Zhang X., Ren S., Sun J. Deep residual learning for image recognition. Proceedings of the 2016 IEEE Conference on Computer Vision and Pattern Recognition (CVPR).

[29] Jolliffe I., Lovric M. (2011). Principal component analysis. International Encyclopedia of Statistical Science.

[30] Yeganeh H., Wang Z. (2012). Objective quality assessment of tone-mapped images. IEEE Trans. Image Process..

[31] Pertuz S., Puig D., Garcia M.A. (2013). Analysis of focus measure operators for shape-from-focus. Pattern Recognit..

[32] Haralick R.M., Shanmugam K., Dinstein I.H. (1973). Textural features for image classification. IEEE Trans. Syst. Man Cybern..

[33] Deng D., Zhang J., Wang T., Su J. (2015). A new algorithm of image quality assessment for photospheric images. Res. Astron. Astrophys..

[34] Krotkov E. (1988). Focusing. Int. J. Comput. Vis..

[35] Wang Z., Bovik A.C., Sheikh H.R., Simoncelli E.P. (2004). Image quality assessment: From error visibility to structural similarity. IEEE Trans. Image Process..

[36] Kovesi P. (1999). Image features from phase congruency. Videre: J. Comput. Vis. Res..

[37] Zhang L., Zhang L., Mou X., Zhang D. (2011). FSIM: A feature similarity index for image quality assessment. IEEE Trans. Image Process..

[38] Cortes C., Vapnik V. (1995). Support-vector networks. Mach. Learn..

[39] Chen T., Guestrin C. XGBoost: A scalable tree boosting system. Proceedings of the 22nd ACM SIGKDD International Conference on Knowledge Discovery and Data Mining.

[40] Breiman L. (2001). Random forests. Mach. Learn..

[41] Lemen J.R., Title A.M., Akin D.J., Boerner P.F., Chou C., Drake J.F., Duncan D.W., Edwards C.G., Friedlaender F.M., Heyman G.F. (2012). The atmospheric imaging assembly (AIA) on the solar dynamics observatory (SDO). Sol. Phys..

